# Comparative *De Novo* transcriptome analysis of the Australian black-lip and Sydney rock oysters reveals expansion of repetitive elements in *Saccostrea* genomes

**DOI:** 10.1371/journal.pone.0206417

**Published:** 2018-10-25

**Authors:** Carmel McDougall

**Affiliations:** Australian Rivers Institute, Griffith University, Nathan, Queensland, Australia; Bigelow Laboratory for Ocean Sciences, UNITED STATES

## Abstract

Ostreid oysters (the ‘true oysters’) represent a large and commercially important family of bivalve molluscs. Several species, such as the Pacific oyster (*Magallana gigas*), the American oyster *(Crassostrea virginica*), the European oyster (*Ostrea edulis*) and the Sydney rock oyster (*Saccostrea glomerata*), are currently farmed at a large scale. However a number of other species may also be suitable for commercial-scale aquaculture. One such species is the ‘black-lip oyster’, a large *Saccostrea* species of uncertain taxonomic affinity found in northern Australia. Here, phylogenetic analysis of the *COI* gene places this oyster within a clade identified in a previous study of Japanese *Saccostrea* species, *‘Saccostrea lineage J’*. To facilitate comparisons between this oyster and the better-studied *S*. *glomerata*, *de novo* transcriptomes were generated from larval stages and adult tissues of both species. Patterns of orthology indicated an expansion of repetitive elements within *Saccostrea* genomes when compared to *M*. *gigas* and *C*. *virginica*, which may be reflected in increased evolutionary rates and/or genome sizes. The generation of high-quality transcriptomes for these two commercially relevant oysters provides a valuable resource for gene identification and comparison of molecular processes in these and other mollusc species.

## Introduction

Aquaculture of ostreid oysters is a significant industry worldwide, with an estimated value of $US6.6 billion per annum (2016 data, [1]). Within Australia, the majority of production is focused on two species, the native Sydney rock oyster (*Saccostrea glomerata*), and the introduced Pacific oyster (*Magallana gigas*, formerly *Crassostrea gigas*) [2]. Production of each of these species is hampered by mass mortality events due to disease outbreaks, and significant effort is being expended towards selective breeding of disease resistant lines for both species [3–6]. An additional suggested course of action is to investigate other native species for aquaculture potential [7], which, depending on the species, may facilitate the establishment of the industry in new coastal regions (for example, the tropics).

A prime candidate for tropical oyster aquaculture in Australia is the ‘black-lip’ oyster, a large *Saccostrea* species currently farmed on a small scale in Bowen, Queensland (John Collison, personal communication) and Darwin, Northern Territory [8]. The taxonomic status of this oyster is poorly defined, and it is variably reported in the literature as ‘*Saccostrea echinata’* [8, 9] or ‘*Striostrea (Parastriostrea) mytiloides’* [2, 10]. Given that unambiguous identification of *Saccostrea* oysters is challenging based on morphology alone [11, 12], and that no molecular data exists for the species, this designation must be treated as tentative at this stage. It is assumed that the species currently farmed is the same as that reported from Magnetic Island, Queensland [9, 13], New Caledonia [14], and Palau [15].

Aquaculture of the black-lip oyster has largely relied on natural catch of juvenile oysters (spat), and the lack of established hatchery protocols for this species is a major impediment to expansion of production. Recent reports indicate that the poor larval survival previously experienced in hatchery trials [9, 14] has largely been overcome, however settlement rates remain low [8]. Current hatchery production protocols have been adapted from those developed for the Sydney rock oyster (*Saccostrea glomerata*) [16], and may not be optimal for black-lip oysters. Differences in settlement processes could be expected given that black-lip oysters are typically found as isolated individuals on mangrove roots and rocks [13, 17], whereas *S*. *glomerata* is found in large aggregations [18]. Settlement of marine pelagobenthic invertebrates is a tightly regulated molecular process, whereby larvae at a particular genetically-determined developmental state (competence) are receptive to particular internal or external cues that initiate metamorphosis [19]. Improved understanding of this process in the black-lip oyster will be critical for the successful hatchery production of this species.

Molecular approaches can likewise be applied to other aspects of oyster husbandry, and have already demonstrated promise for the improvement of *S*. *glomerata* production. A selective breeding program for faster growth and disease resistance was initiated in New South Wales in 1990 [4, 20, 21]. Genes encoding anti-oxidant enzymes were shown to be differentially expressed between disease-resistant and wild-type oysters, highlighting the potential for marker-assisted selection [22]. Molecular techniques have also yielded tools for the manipulation of broodstock condition, a development of critical importance for the hatchery production of selected animals [23]. Given that the black-lip and *S*. *glomerata* belong to the same genus it is possible that some of the molecular techniques developed may be directly transferrable between the species.

The development of these molecular approaches relies on the availability of sequence data for the species of interest. A number of transcriptome studies have been performed for *S*. *glomerata*, investigating gene expression in different tissues and under different environmental conditions and stressors [23–28]. None of these transcriptomic studies have incorporated larval samples, therefore genes expressed exclusively in larval stages will not be identified in these transcriptomes. Aside from *S*. *glomerata*, transcriptome data exists only for one other *Saccostrea* species, *S*. *palmula* [29], limiting capacity for comparative analyses between taxa.

This study presents comprehensive transcriptomes derived from larval stages and adult tissues of both the black-lip and Sydney rock oysters. The transcriptomes were deemed to be of high quality, based on assembly completeness and comparison of universal benchmarking statistics against whole genome assemblies of *M*. *gigas* and *Crassostrea virginica*. Patterns of orthology were compared between the *Saccostrea* transcriptomes generated here and predicted proteins from the Crassostreinae (*M*. *gigas* and *C*. *virginica*) genomes. This revealed that the *Saccostrea* lineages possess a larger repertoire of repetitive elements, particularly within the *LINE*, *SINE* and *Penelope* classes. The transcriptomes presented here will provide a valuable resource not only for improvement of oyster production, but also for investigation into the evolution of life-history traits in oysters.

## Materials and methods

### Animal sources, husbandry, and sample collection

All samples were taken during commercial hatchery runs conducted at Aquafarms Queensland Pty Ltd, Hervey Bay, Australia. Adult black-lip and *S*. *glomerata* were supplied from commercial oyster farms in Bowen, Queensland, and Port Stephens, New South Wales, respectively. Oyster spawning and larval culture followed the methods outlined in [16]. Briefly, oysters were induced to spawn via temperature and salinity treatments. Individuals that had begun spawning were removed and kept separate until spawning was complete, and eggs and sperm from all individuals pooled separately prior to fertilisation. Larvae were stocked in 5,000 L tanks containing aerated filtered sea water (FSW) at an initial concentration of 19 (black-lip) or 26 (*S*. *glomerata*) larvae mL^-1^, and were maintained at 27°C ± 2°C (black-lip) or 25°C ± 2°C (*S*. *glomerata*). Larvae were drained on to mesh screens and transferred to a clean 5,000L tank daily, and were periodically screened to remove the slowest growers. Feeding was initiated at approximately 16 hours post fertilisation (hpf), and consisted of *Pavlova lutheri*, *Tisochrysis lutea*, *Chaetoceros calcitrans* and *Chaetoceros muelleri*, at varying concentrations depending on larval age. Settlement induction was performed using epinephrine bitartrate once larvae had reached the pediveliger stage and were able to be retained on a 200 μm mesh screen (day 24 for the black-lip, day 21 for *S*. *glomerata*).

Embryos and larvae (~50 per stage) were sampled at least once daily, with more frequent sampling within the first 24 hours and at settlement. Small (<0.25 mg) samples were also taken from various tissues from two *S*. *glomerata* and three black-lip adults. All samples were taken in to microcentrifuge tubes containing 1ml RNAlater (Sigma), stored at 4°C for 24 hours, and then -20°C until RNA extraction. Details of all samples are provided in S1 Table.

### DNA extraction and *COI* sequencing of the black-lip

A small piece of adductor muscle (~0.25 mg) was dissected from three adult black-lip individuals for DNA extraction. Extractions were performed using a standard NETS/phenol chloroform protocol. PCR amplification of a partial *COI* sequence was performed using the primers LCO1490 and HCO2198 [30], 10ng of DNA template, and *Taq* polymerase (NEB) with the following cycling conditions: an initial denaturation at 94°C for two minutes, 30 cycles of 94°C for 30 seconds, 48°C for 30 seconds, and 68°C for 45 seconds, followed by a final extension of 68°C for 10 minutes. Resulting products were gel purified and submitted to the Australian Genome Research Facility, Brisbane, for Sanger sequencing in both directions using the PCR primers.

### Phylogenetic analysis

Resultant *COI* sequences were trimmed to remove the primer and aligned with other partial ostreid *COI* sequences (downloaded from NCBI) using the program Aliview [31]. The alignment was manually trimmed to the minimal length of the majority of the records (538 bp) and shorter sequences were removed (S1 Alignment). Phylogenetic analysis was performed using RAxML 8.2.11 [32] using the GTR (general time reversible) substitution model with the CAT model of rate heterogeneity and 100 nonparametric bootstrap replicates. Phylogenetic trees were visualised using FigTree [33].

### Transcriptome sequencing, assembly, and quality assessment

For each species, RNA extractions from embros/larvae and adult tissues were performed separately and pooled prior to preparation of sequencing libraries. A minimum of 20 individual embryos or larvae from each sampled stage were pooled in to three separate extractions to maximise coverage of the entire larval timecourse. Samples were homogenised using glass pestles in 1 mL of TRI Reagent (Sigma) and extractions performed as per the manufacturer’s instructions, using 1-bromo-3-chloropropane for phase separation. Precipitation of RNA was performed using 0.25 mL of isopropanol and 0.25 mL of high salt precipitation solution (0.8 M sodium citrate and 1.2 M sodium chloride). RNA was shipped to Macrogen (Seoul, Korea) and assessed for quality on an Agilent 4200 Tapestation High Sensitivity ScreenTape before library preparation using a TruSeq Stranded mRNA LT Sample Prep Kit. Libraries were sequenced on a HiSeq 2500 to generate ~80,000 100 bp paired-end reads. Quality of the resulting data was assessed using FastQC 0.11.3 [34].

Transcriptome assembly was performed using Trinity 2.4.0 [35], with quality trimming via Trimmomatic and without normalisation of reads. Transcriptome quality assessment was performed by 1) determining the level of representation of reads within the assembly by mapping using Bowtie2 2.0.2 [36], and 2) determining the proportion of full-length transcripts via BLAST+ 2.3.0 [37] alignment of sequences in the SwissProt non-redundant database [38] against the assemblies, both as outlined in the Trinity documentation. Analysis of assembly completeness was performed using BUSCO v3 [39] and the metazoa_odb9 dataset (created 13/02/2016), analysing open reading frames identified within transcripts by TransDecoder 5.3.0 [35]. Results for the transcriptomes generated here were compared against whole genome data from the oysters *M*. *gigas* [40] (PRJNA276446) and *C*. *virginica* [41](PRJNA379157).

Transcriptome annotation was conducted via sequence homology searching of transcripts against the Swissprot database by BLAST [42], the PFAM database [43] by hmmscan [44], and association with Gene Ontology (GO) terms [45], all within the Trinotate 3.1.1 workflow (https://github.com/Trinotate/Trinotate.github.io/wiki) [46].

### Orthology and enrichment analysis

To reduce sequence redundancy present within the transcriptomes, TransDecoder was first used to identify the longest potential open reading frame per transcript using the -single_best_orf command. The dataset was further filtered to retain only the single longest isoform per Trinity ‘gene’. These predicted protein datasets, generated for each species, were then analysed along with predicted proteins from the *M*. *gigas* and *C*. *virginica* genomes using Orthofinder 2.2.3 [47] to identify groups of potentially orthologous proteins and their corresponding genes. From this analysis, genes within the *Saccostrea-*specific orthogroups were analysed for overrepresentation of ‘biological process’ GO categories using a hypergeometric test within the BiNGO plugin [48] of Cytoscape [49]. The analysis was performed in both directions, i.e., using the *S*. *glomerata* transcripts from the *Saccostrea*-specific orthogroups against the complete annotation of the *S*. *glomerata* predicted protein dataset as a reference, and again using the *S*. *lin*. *J* transcripts against the complete annotation of the *S*. *lin*. *J* dataset. Enrichments with an adjusted p-value of less than 0.01 (Benjamini and Hochberg FDR correction) in both species were deemed to be significant.

### Analysis of repetitive elements

Analysis of repetitive element content was performed using RepeatMasker 4.0.7 [50] by aligning sequences against RepBase (RepBase-20170127)[51]. The analysis was performed for *S*. *glomerata* and the black-lip using the reduced ‘single longest isoform per gene’ dataset outlined above, and all predicted genes from the *M*. *gigas* and *C*. *virginica* genomes. The parameters for the analysis were: -q -species 'fungi/metazoa group’.

## Results and discussion

### Description and identification of the ‘black-lip’ oyster

**Fig 1:**
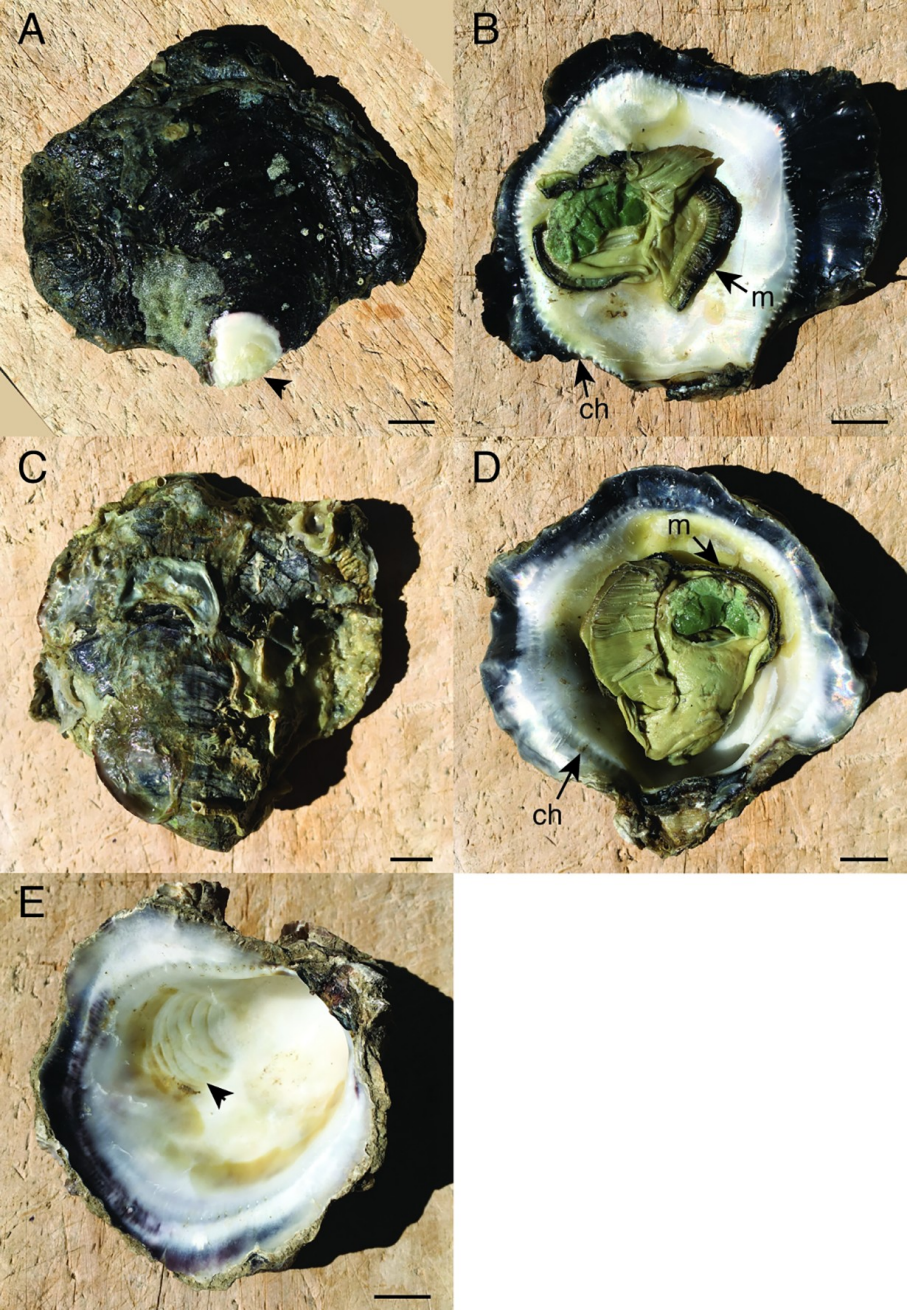
Morphology of the black-lip oyster. The sample has been stored in ethanol, causing some shift in colour within the internal soft tissues. A. Upper surface of the right valve, showing intense dark pigmentation. The outer shell layer has been chipped away at the umbo (arrowhead). B. Inner surface of the right valve. The dark outer shell margin is sharply separated from the white internal surface by large, obvious chomata (ch). The mantle edge is darkly pigmented (m). C. Outer surface of the deeply-cupped left valve. D. Inner surface of the left valve, showing a dark outer shell margin and obvious chomata, and dark pigmentation in the mantle E. Inner surface of the left valve of a second specimen, with soft tissues removed to display the adductor scar (arrowhead). Scale bar = 10mm.

The black-lip oysters used in this study were farmed adults sourced from wild-caught spat in Bowen, Queensland, Australia. The oysters were large (70–84 mm in diameter), and characterised by a dark outer surface of the right (upper) valve, a distinct black margin around the inner surface of the right valve, thick shell, prominent chomata, and a dark mantle margin (“black-lip”) (Fig 1).

**Fig 2:**
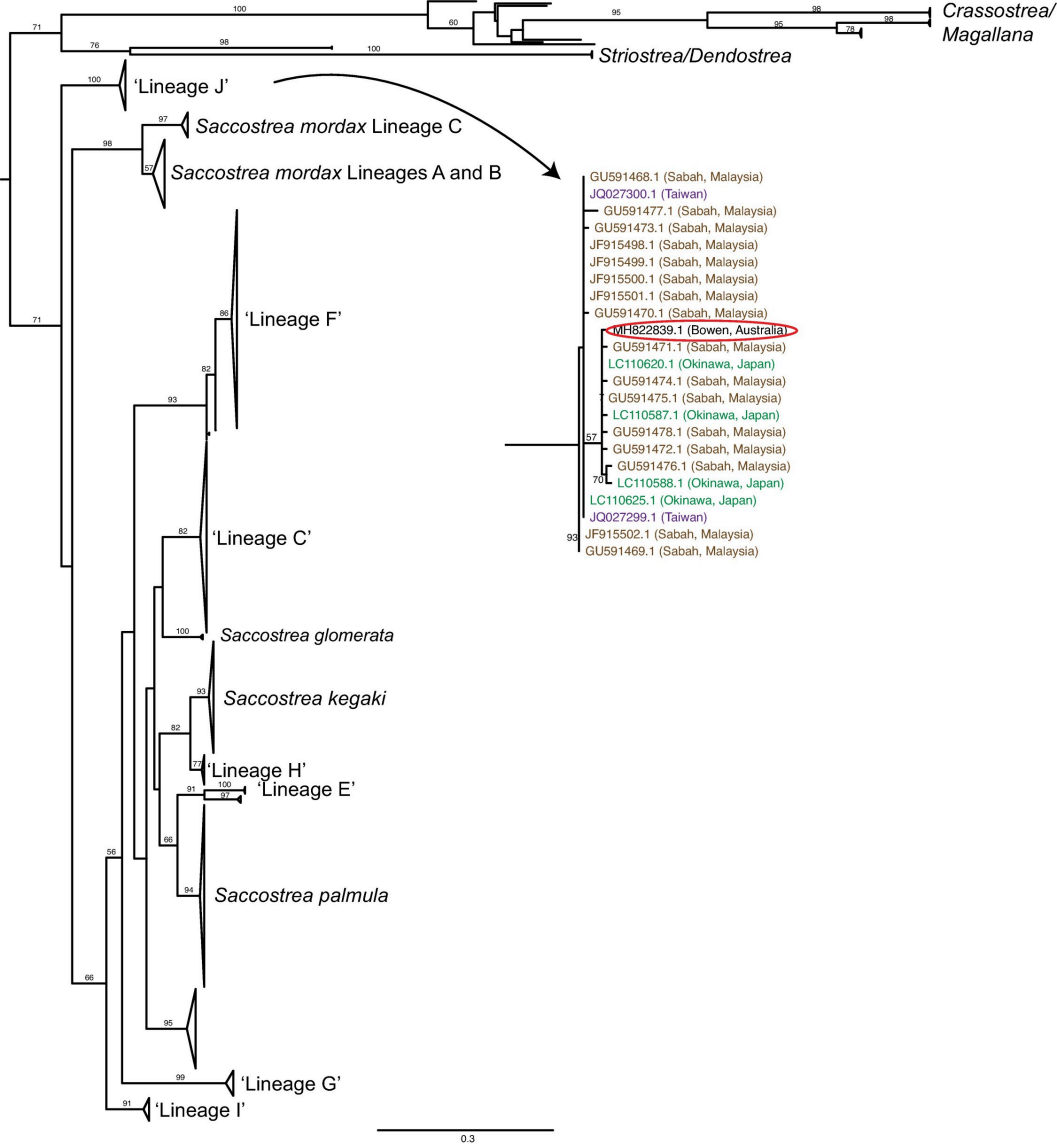
Maximum likelihood phylogenetic analysis of *Saccostrea COI* sequences. Bootstrap values >50 are given on branches, and the scale bar indicates the number of substitutions per site. The clade containing *Striostrea*, *Dendostrea*, *Magallana* and *Crassostrea COI* sequences is used as an outgroup. Lineages have been designated (where possible) following Lam and Morton [11] and Sekino and Yamashita [12]. The Bowen black-lip *COI* sequence (circled in inset) falls within ‘Lineage J’ with strong support.

A 649 bp fragment of the *COI* gene was sequenced from three adult black-lip oysters. All three sequences were identical (NCBI accession MH822839). Phylogenetic analysis recovered major clades largely congruent with that of previous studies [11, 12, 52] (Fig 2, S1 Fig), placing the black-lip oyster within ‘Lineage J’ as nominated by Sekino and Yamashita [12]. The black-lip is therefore designated as *‘Saccostrea lin*. *J’* henceforth. Other oysters within lineage J were collected from Japan (Okinawa), Malaysia (Sabah) and Taiwan, suggesting a broad tropical Indo-Pacific distribution for this lineage. In both this analysis and that of Sekino and Yamashita [12] lineage J falls as a sister clade to all other sequenced *Saccostrea* lineages, albeit with low support.

### Transcriptome sequencing and assembly

To obtain representative transcriptomes for both *S*. *glomerata* and *S*. *lin*. *J*, RNA from larval stages and adult tissues were pooled prior to library generation, high-throughput sequencing, and transcriptome assembly. Raw sequence files and assembled transcripts have been deposited in NCBI under BioProject PRJNA487836. Although similar numbers of raw reads (~80 million) were obtained for both libraries, significantly more assembled transcripts were obtained for *S*. *glomerata* than *S*. *lin*. *J* (Table 1). Despite this, the median contig length and contig N50 were higher for the *S*. *lin*. *J* assembly, indicating that the higher transcript count may reflect a more fragmented assembly for *S*. *glomerata*. Overall, these quality statistics are similar to those of other recent bivalve transcriptomes sequenced using similar techniques [26, 53].

**Table 1:** Assembly statistics for *S*. *lin*. *J* and *S*. *glomerata* transcriptomes.

	*S*. *lin*. *J*	*S*. *glomerata*
Raw reads (paired-end)	80,450,850	79,209,388
Total number of Trinity ‘genes’	120621	180564
Total number of assembled transcripts	216545	375022
GC content (%)	38.30	38.73
Contig N50	1508	1109
Median contig length (nt)	446	400
Total assembled bases (nt)	185,573,484	266,516,557

### Transcriptome quality assessment and annotation

Alignment of raw reads back to the Trinity assemblies revealed that 91.76 and 85.05 percent of paired reads aligned concordantly to the *de novo* transcriptomes for *S*. *lin*. *J* and *S*. *glomerata*, respectively, indicating a high read representation within each assembly. The degree to which assembled transcripts were likely to be full length was assessed by aligning each sequence in the Swissprot non-redundant database against the transcriptome via BLAST. For the 12358 Swissprot sequences with BLAST hits in the *S*. *lin*. *J* assembly, 4150 (33.6%) had at least one transcript that aligned along the entire sequence, whereas 3465 (23.9%) of the Swissprot sequences appeared to be represented by a full-length transcript in the *S*. *glomerata* assembly.

Transcriptome completeness was assessed using the Benchmarking Universal Single-Copy Ortholog (BUSCO) assessment tool [39]. *De novo* transcriptomes were compared against whole genome protein data from *M*. *gigas* and *C*. *virginica*. The *S*. *glomerata* transcriptome lacks 8 BUSCOs, equal to the number missing from the *M*. *gigas* genome and less than that missing from *C*. *virginica* (Table 2). Even fewer BUSCOs (4) were lacking from the *S*. *lin*. *J* transcriptome. One BUSCO, EOG091G0GA7, was missing from all four oyster datasets, indicating that this gene (encoding a putative N-6 adenine-specific DNA methyltransferase) may have been lost from the ostreid lineage. Overall, these results indicate that the transcriptomes reported here provide good coverage of the total gene content of these species.

**Table 2:** BUSCO results for both *Saccostrea* species compared to Crassostreinae whole-genome data.

	*S*. *lin*. *J*	*S*. *glomerata*	*M*. *gigas*	*C*. *virginica*
Complete BUSCOs	968	924	963	960
Complete and single-copy BUSCOs	534	420	685	538
Complete and duplicated BUSCOs	434	504	278	422
Fragmented BUSCOs	6	46	7	2
Missing BUSCOs	4	8	8	16

### Analysis of orthologous sequences

The high degree of transcript redundancy within the transcriptome datasets indicated via BUSCO analysis necessitated filtering of sequence data with the aim of retaining only one transcript per gene for downstream orthology and enrichment analysis. To achieve this, the longest open reading frame was selected per Trinity ‘isoform’, followed by selection of the longest isoform per Trinity ‘gene’. It should be noted that filtering by this method does remove some valid sequences, as reflected by a larger number of missing BUSCOs for these datasets (7 and 33 missing BUSCOs for *S*. *lin*. *J* and *S*. *glomerata*, respectively).

**Fig 3:**
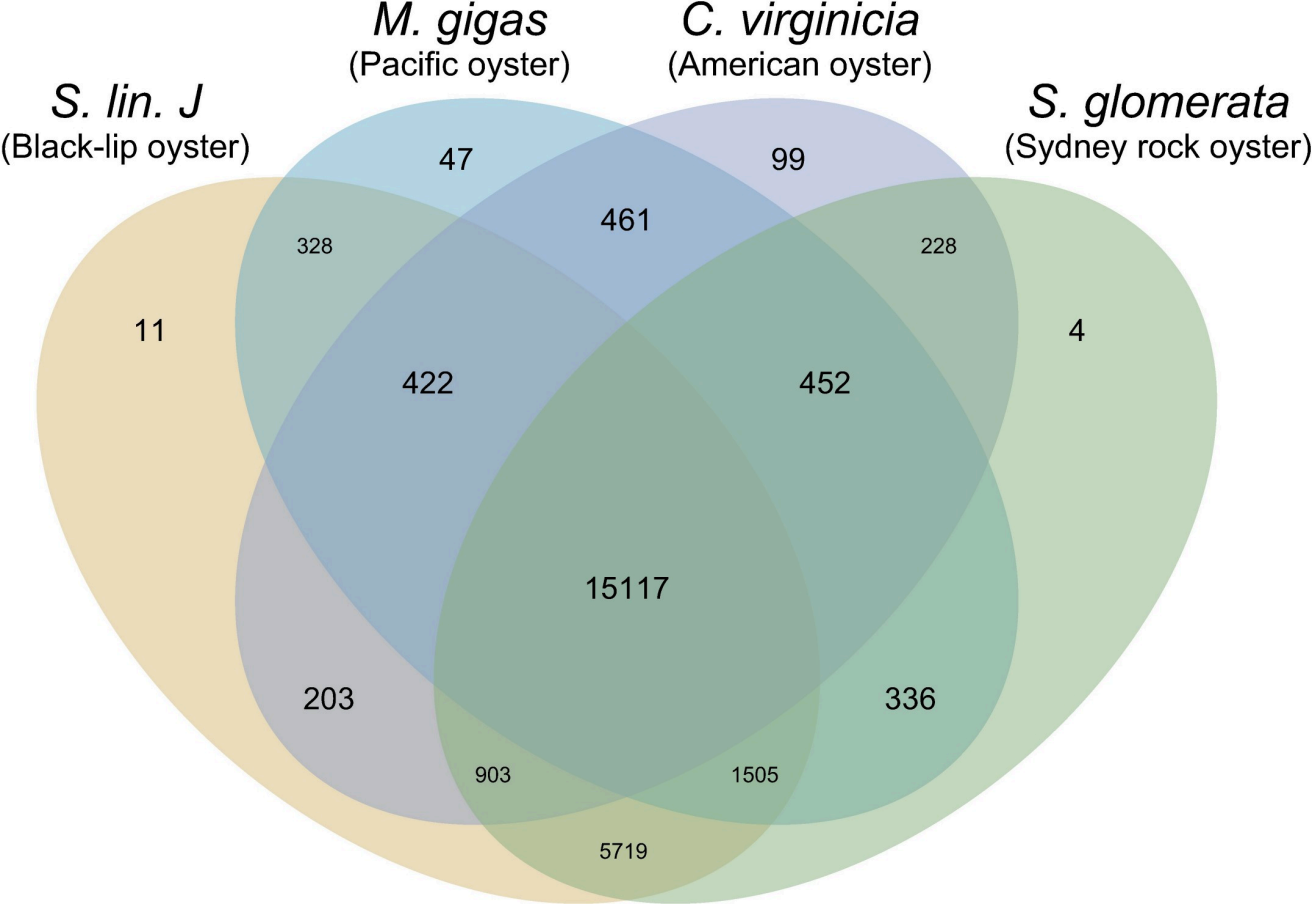
Patterns of gene orthology in oyster species. Number of orthogroups shared between *Saccostrea lineage J*, *Saccostrea glomerata*, *Magallana gigas*, and *Crassostrea virginica*. 15117 orthogroups are shared between all four species, and 5719 orthogroups are shared exclusively between the two *Saccostrea* species.

Orthology analysis performed on this filtered dataset revealed that 15,117 orthogroups (inferred set of genes descended from a single ancestral gene) were shared between all four species (Fig 3). An additional 5,719 orthogroups were shared exclusively by the two *Saccostrea* species, whereas only 461 were shared exclusively by *M*. *gigas* and *C*. *virginica*. This large differential was unexpected and possibly reflected the differences in sequencing methodology (*i*.*e*., transcriptome *vs* whole genome) for these species, rather than a true biological difference. To investigate this further, GO-term enrichment was performed to gain insight into the putative functions of orthogroups shared exclusively between the two *Saccostrea* species.

### Enrichment analysis

Potential functional enrichment within the *Saccostrea*-specific orthogroups identified above was investigated by assessing the representation of GO categories (based upon Swissprot BLAST results) within this subset of transcripts against the whole transcriptome annotation. 43 GO categories were enriched within the *Saccostrea*-specific orthogroups using an adjusted p-value of less than 0.01 (Table 3). Of these, 11 represented terminal (non-parent) GO terms.

**Table 3:** GO categories enriched in *Saccostrea*-specific orthogroups.

GO Term	Description	Terminal GO term	*S*. *lin*. *J* P value (adj)	*S*. *glomerata* P value (adj)
0006278	RNA-dependent DNA biosynthetic process		3.61E-17	8.46E-23
0015074	DNA integration	Y	2.94E-16	5.17E-15
0032196	transposition		2.94E-16	5.04E-19
0015969	guanosine tetraphosphate metabolic process	Y	1.74E-12	4.87E-32
0034035	purine ribonucleoside bisphosphate metabolic process		1.21E-11	2.13E-30
0006259	DNA metabolic process		1.34E-11	1.26E-13
0071897	DNA biosynthetic process		1.78E-11	5.62E-17
0006313	transposition, DNA-mediated	Y	9.23E-11	1.11E-11
0034032	purine nucleoside bisphosphate metabolic process		6.10E-10	6.83E-27
0033875	ribonucleoside bisphosphate metabolic process		6.10E-10	6.83E-27
0033865	nucleoside bisphosphate metabolic process		6.10E-10	6.83E-27
0006310	DNA recombination		4.74E-09	5.19E-13
1901068	guanosine-containing compound metabolic process		1.15E-08	8.46E-23
0046128	purine ribonucleoside metabolic process		7.01E-08	3.16E-21
0042278	purine nucleoside metabolic process		1.04E-07	7.58E-21
0009119	ribonucleoside metabolic process		1.26E-06	8.70E-19
0045599	negative regulation of fat cell differentiation	Y	5.13E-05	2.82E-05
0090084	negative regulation of inclusion body assembly	Y	6.26E-05	3.84E-05
0009116	nucleoside metabolic process		7.24E-05	1.04E-16
0048147	negative regulation of fibroblast proliferation	Y	8.86E-05	5.70E-04
0090083	regulation of inclusion body assembly		1.67E-04	5.17E-05
0070373	negative regulation of ERK1 and ERK2 cascade	Y	1.76E-04	1.50E-04
0007155	cell adhesion		1.82E-04	5.37E-04
0022610	biological adhesion		2.07E-04	6.32E-04
0032197	transposition, RNA-mediated		2.24E-04	8.49E-06
1901657	glycosyl compound metabolic process		2.24E-04	1.01E-14
1901017	negative regulation of potassium ion transmembrane transporter activity		3.33E-03	6.90E-05
0045760	positive regulation of action potential		3.79E-03	2.59E-04
1903817	negative regulation of voltage-gated potassium channel activity		3.79E-03	1.19E-04
1901380	negative regulation of potassium ion transmembrane transport		3.88E-03	1.50E-04
0001171	reverse transcription		4.65E-03	1.72E-05
0032199	reverse transcription involved in RNA-mediated transposition	Y	4.65E-03	1.72E-05
1902259	regulation of delayed rectifier potassium channel activity		5.62E-03	1.19E-04
0009150	purine ribonucleotide metabolic process		6.08E-03	2.58E-11
0045598	regulation of fat cell differentiation		7.34E-03	3.00E-03
0006163	purine nucleotide metabolic process		7.34E-03	4.90E-11
0043267	negative regulation of potassium ion transport		7.34E-03	3.21E-04

**Fig 4:**
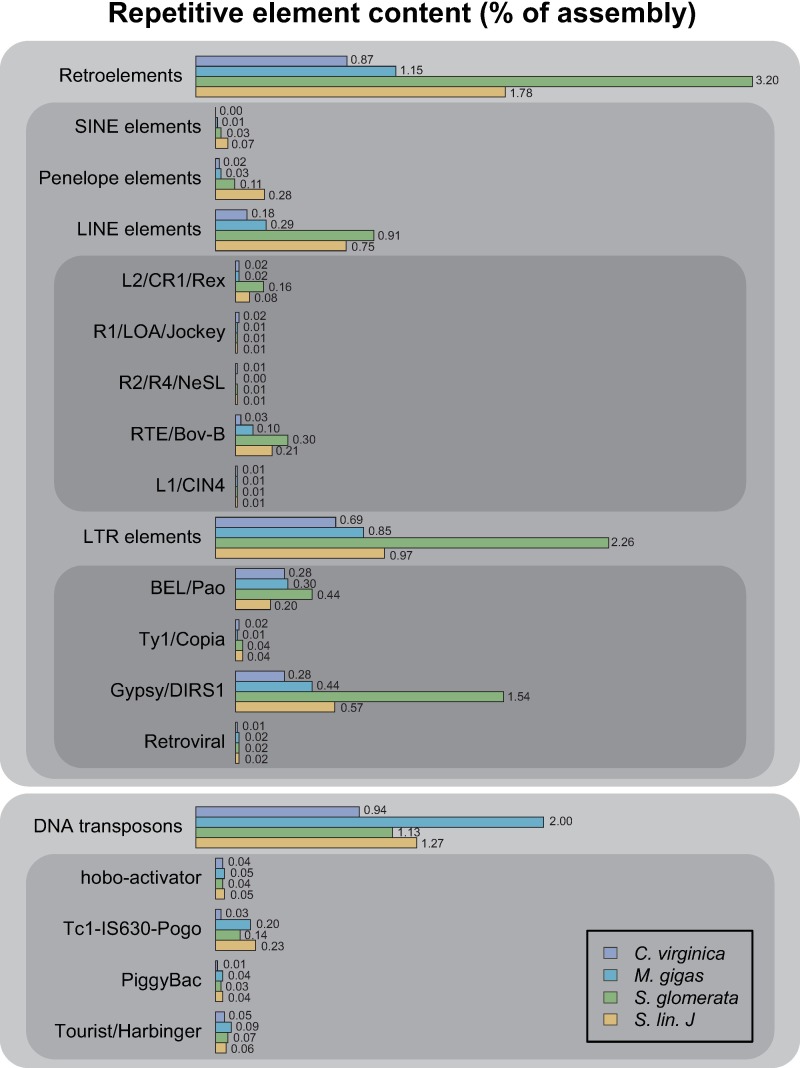
Repetitive element content of oyster genomes as assessed by searches against RepBase. *Saccostrea* species possess a greater proportion of retroelements within their genomes, particularly within the *SINE*, *LINE*, and *Penelope* classes. Additional expansion of the *Gypsy/DIRS1 LTR* class is evident in *S*. *glomerata*.

A number of these enriched terms are associated with repetitive elements (e.g., DNA integration, DNA-mediated transposition, RNA-mediated transposition), indicating the potential expansion of these elements in *Saccostrea* species in relation to *M*. *gigas* and *C*. *virginica* genomes. To investigate this further, RepeatMasker was used to identify and classify transposable elements in each of the four datasets. This analysis detected higher proportions of retroelements in *Saccostrea* lineages, particularly within *SINE*, *Penelope*, and *LINE* classes (Fig 4). *S*. *glomerata* had the highest overall proportion of repetitive elements (3.20%), and appears to have undergone an additional expansion of the Gypsy/DIRS1 class of LTR elements. The total proportion of repetitive element content within *Saccostrea* genomes is likely to be even higher than that reported here, as transcriptomics can only reveal elements that are transcriptionally active. As repetitive element content is positively correlated with genome size [54], these results may also indicate larger genomes for *Saccostrea* species when compared to that of *C*. *virginica* (estimated at 675 Mb, [55]) and *M*. *gigas* (estimated between 545 and 637 Mb, [40]). Alternatively, the higher proportion of repetitive elements found in *Saccostrea* genomes may reflect an underestimation of repetitive element content in *C*. *virginica* and *M*. *gigas* genomes, as masking of repetitive regions prior to gene prediction in whole-genome annotation (e.g., page 18 Supplementary Information in reference [40]) may preclude accurate identification.

Transposable elements are major sources of genetic variation within genomes (reviewed in [56]). The composition of transposable elements can vary even between closely related species, likely the result of differential timing and rates of proliferation events, accumulation of mutations, horizontal transfer, and differential rates of transposable element removal. In some cases, differential transposable element proliferation has been implicated as a major driver of the speciation process itself (reviewed in [57]). *Penelope* elements, in particular, are associated with chromosomal rearrangements and mutations in hybrids of different genetic populations (hybrid dysgenesis), and in the induction of other transposable elements [58]. *Penelope* expansions have been reported in other bivalves, for example, in *Mytilus galloprovincialis* [59] and *Ostrea edulis* [60], and may therefore have major implications for the evolution of bivalve genomes.

## Conclusion

This study presents high-quality transcriptomes derived from embryos, larvae, and adult tissues of two *Saccostrea* species, *S*. *glomerata* and *S*. *lineage J*. Analysis of gene orthology patterns between these transcriptomes and whole genome data from the oysters *Magallana gigas* and *Crassostrea virginica* demonstrated an expansion of repetitive elements within the *Saccostrea* lineage, possibly revealing an important mechanism for the generation of genetic diversity within these genomes. The transcriptomes developed in this study extend the molecular data available for ostreid bivalves, and provide a valuable resource for future comparative genomics of these commercially important species.

## Supporting information

S1 Alignment Trimmed sequence alignment used for phylogenetic analysis, in FASTA format. (FA)

S1 Fig Uncollapsed version of the phylogenetic tree presented in Fig 2. (PDF)

S1 Table Details of samples taken for gene expression analyses. (XLSX)

## References

[1] FAO. FAO Yearbook. Fishery and aquaculture statistics 2016. Rome: 2018.

[2] Nell JA. The history of oyster farming in Australia. Marine Fisheries Review. 2001;63(3): 14–25.

[3] Green TJ, Raftos D, O'Connor W, Adlard RD, Barnes AC. Disease prevention strategies for QX disease (*Marteilia sydneyi*) of Sydney rock oysters (*Saccostrea glomerata*). J Shellfish Res. 2011;30(1): 47–53. 10.2983/035.030.0108

[4] Dove MC, Nell JA, O'Connor WA. Evaluation of the progeny of the fourth-generation Sydney rock oyster *Saccostrea glomerata* (Gould, 1850) breeding lines for resistance to QX disease (*Marteilia sydneyi*) and winter mortality (*Bonamia roughleyi*). Aquac Res. 2012;44(11): 1791–800. 10.1111/are.12012

[5] Paul-Pont I, Dhand NK, Whittington RJ. Influence of husbandry practices on OsHV-1 associated mortality of Pacific oysters *Crassostrea gigas*. Aquaculture. 2013;412–413: 202–14. 10.1016/j.aquaculture.2013.07.038

[6] Ugalde SC, Preston J, Ogier E, Crawford C. Analysis of farm management strategies following herpesvirus (OsHV-1) disease outbreaks in Pacific oysters in Tasmania, Australia. Aquaculture. 2018;495: 179–86. 10.1016/j.aquaculture.2018.05.019

[7] Davis J. A national industry response to Pacific oyster mortality syndrome (POMS). Agribusiness Tasmania, Launceston: 2016.

[8] Nowland SJ, O'Connor W, Southgate PC. Embryonic, larval, and early postlarval development of the tropical black-lip rock oyster *Saccostrea echinata*. J Shellfish Res. 2018;37(1): 1–5. 10.2983/035.037.0100

[9] Southgate PC, Lee PS. Hatchery rearing of the tropical blacklip oyster *Saccostrea echinata* (Quoy and Gaimard). Aquaculture. 1998;169: 275–81.

[10] Munksgaard NC, Burchert S, Kaestli M, Nowland SJ, O'Connor W, Gibb KS. Cadmium uptake and zinc-cadmium antagonism in Australian tropical rock oysters: Potential solutions for oyster aquaculture enterprises. Mar Pollut Bull. 2017;123: 47–56. 10.1016/j.marpolbul.2017.09.031 28938999

[11] Lam K, Morton B. Morphological and mitochondrial-DNA analysis of the Indo-West Pacific rock oysters (Ostreidae: *Saccostrea* species). J Mollus Stud. 2006;72(3): 235–45. 10.1093/mollus/eyl002

[12] Sekino M, Yamashita H. Mitochondrial and nuclear DNA analyses of *Saccostrea* oysters in Japan highlight the confused taxonomy of the genus. J Mollus Stud. 2016;82(4): 492–506. 10.1093/mollus/eyw022

[13] Bryson RK. Tropical oyster farming—a valuable new industry. Aust Fish. 1977;36(11): 2-6–15.

[14] Coeroli M, De Gaillande D, Landret J. Recent innovations in cultivation of molluscs in French Polynesia. Aquaculture. 1984;39: 45–67.

[15] Braley R. Mariculture potential of introduced oysters *Saccostrea cucullata tuberculata* and *Crassostrea echinata*, and a histological study of reproduction of *C*. *echinata*. Aust J Mar Freshwater Res. 1984;35: 129–41.

[16] O'Connor WA, Dove M, Finn B, O'Connor S. Manual for production of Sydney rock oysters (*Saccostrea glomerata*) Fisheries Research Report Series. Port Stephens Fisheries Centre, NSW Department of Primary Industries; New South Wales, Australia: 2008.

[17] Glude JB. The applicability of recent innovations to mollusc culture in the western Pacific islands. Aquaculture. 1984;39(1–4): 29–43. 10.1016/0044-8486(84)90257-6

[18] Krassoi FR. Population ecology of the Sydney rock oyster Saccostrea commercialis and the Pacific oyster Crassostrea gigas in a New South Wales estuary. PhD Thesis, University of Technology, Sydney. 2001.

[19] Hadfield MG, Carpizo-Ituarte EJ, del Carmen K, Nedved BT. Metamorphic competence, a major adaptive convergence in marine invertebrate larvae. Am Zool. 2001;41(5): 1123–31. 10.1093/icb/41.5.1123

[20] Nell JA, Smith IR, McPhee CC. The Sydney rock oyster *Saccostrea glomerata* (Gould 1850) breeding programme: progress and goals. Aquac Res. 2000;31(1): 45–9. 10.1046/j.1365-2109.2000.00387.x

[21] Nell JA, Perkins B. Evaluation of progeny of fourth generation Sydney rock oyster *Saccostrea glomerata* (Gould, 1850) breeding lines. Aquac Res. 2005;36(8): 753–7. 10.1111/j.1365-2109.2005.01279.x

[22] Green TJ, Dixon TJ, Devic E, Adlard RD, Barnes AC. Differential expression of genes encoding anti-oxidant enzymes in Sydney rock oysters, *Saccostrea glomerata* (Gould) selected for disease resistance. Fish Shellfish Immunol. 2009;26(5): 799–810. 10.1016/j.fsi.2009.03.003 19332130

[23] In VV, Ntalamagka N, O’Connor W, Wang T, Powell D, Cummins SF, et al Reproductive neuropeptides that stimulate spawning in the Sydney rock oyster (*Saccostrea glomerata*). Peptides. 2016;82: 109–19. 10.1016/j.peptides.2016.06.007 27328253

[24] Goncalves P, Jones DB, Thompson EL, Parker LM, Ross PM, Raftos DA. Transcriptomic profiling of adaptive responses to ocean acidification. Mol Ecol. 2017;26(21): 5974–88. 10.1111/mec.14333 28833825

[25] Ertl NG, O'Connor WA, Brooks P, Keats M, Elizur A. Combined exposure to pyrene and fluoranthene and their molecular effects on the Sydney rock oyster, *Saccostrea glomerata*. Aquat Toxicol. 2016;177: 136–45. 10.1016/j.aquatox.2016.05.012 27286571

[26] Ertl NG, O’connor WA, Papanicolaou A, Wiegand AN, Elizur A. Transcriptome analysis of the Sydney rock oyster, *Saccostrea glomerata*: Insights into molluscan immunity. PLoS ONE. 2016;11(6): e0156649 10.1371/journal.pone.0156649 27258386 PMC4892480

[27] Ertl NG, O’Connor WA, Wiegand AN, Elizur A. Molecular analysis of the Sydney rock oyster (*Saccostrea glomerata*) CO_2_ stress response. Climate Change Responses. 2016;3(1): 6 10.1186/s40665-016-0019-y

[28] Hook SE, Johnston EL, Nair S, Roach AC, Moncuquet P, Twine NA, et al Next generation sequence analysis of the transcriptome of Sydney rock oysters (*Saccostrea glomerata*) exposed to a range of environmental stressors. Mar Genomics. 2014;18: 109–11. 10.1016/j.margen.2014.08.003 25151890

[29] Lemer S, González VL, Bieler R, Giribet G. Cementing mussels to oysters in the pteriomorphian tree: a phylogenomic approach. Proc R Soc B. 2016;283(1833). 10.1098/rspb.2016.0857 27358369 PMC4936043

[30] Folmer O, Black M, Hoeh W, Lutz R, Vrijenhoek R. DNA primers for amplification of mitochondrial cytochrome c oxidase subunit I from diverse metazoan invertebrates. Mol Mar Biol Biotechnol. 1994;3(5): 294–9. 7881515

[31] Larsson A. AliView: a fast and lightweight alignment viewer and editor for large data sets. Bioinformatics. 2014;30(22): 3276–8. 10.1093/bioinformatics/btu531 25095880 PMC4221126

[32] Stamatakis A. RAxML-VI-HPC: maximum likelihood-based phylogenetic analyses with thousands of taxa and mixed models. Bioinformatics. 2006;22(21): 2688–90. 10.1093/bioinformatics/btl446 16928733

[33] Rambaut A. FigTree 1.1.1 ed Edinburgh: University of Edinburgh; 2006.

[34] Andrews S. FastQC A quality control tool for high throughput sequence data. Available from: http://www.bioinformatics.babraham.ac.uk/projects/fastqc/.

[35] Haas BJ, Papanicolaou A, Yassour M, Grabherr M, Blood PD, Bowden J, et al *De novo* transcript sequence reconstruction from RNA-seq using the Trinity platform for reference generation and analysis. Nat Protoc. 2013;8(8): 1494–512. 10.1038/nprot.2013.084 23845962 PMC3875132

[36] Langmead B, Trapnell C, Pop M, Salzberg SL. Ultrafast and memory-efficient alignment of short DNA sequences to the human genome. Genome Biol. 2009;10(3): R25 10.1186/gb-2009-10-3-r25 19261174 PMC2690996

[37] Camacho C, Coulouris G, Avagyan V, Ma N, Papadopoulos J, Bealer K, et al BLAST+: architecture and applications. BMC Bioinformatics. 2009;10: 421 10.1186/1471-2105-10-421 20003500 PMC2803857

[38] The UniProt Consortium. UniProt: the universal protein knowledgebase. Nucleic Acids Res. 2017;45(D1): D158–D69. 10.1093/nar/gkw1099 27899622 PMC5210571

[39] Waterhouse RM, Seppey M, Simão FA, Manni M, Ioannidis P, Klioutchnikov G, et al BUSCO Applications from Quality Assessments to Gene Prediction and Phylogenomics. Mol Biol Evol. 2017;35(3): 543–8. 10.1093/molbev/msx319 29220515 PMC5850278

[40] Zhang G, Fang X, Guo X, Li L, Luo R, Xu F, et al The oyster genome reveals stress adaptation and complexity of shell formation. Nature. 2012;490(7418): 49–54. 10.1038/nature11413 22992520

[41] Gomez-Chiarri M, Warren WC, Guo X, Proestou D. Developing tools for the study of molluscan immunity: The sequencing of the genome of the eastern oyster, *Crassostrea virginica*. Fish Shellfish Immunol. 2015;46(1): 2–4. 10.1016/j.fsi.2015.05.004 25982405

[42] Altschul SF, Gish W, Miller W, Myers EW, Lipman DJ. Basic local alignment search tool. J Mol Biol. 1990;215(3): 403–10. 10.1016/S0022-2836(05)80360-2 2231712

[43] Finn RD, Coggill P, Eberhardt RY, Eddy SR, Mistry J, Mitchell AL, et al The Pfam protein families database: towards a more sustainable future. Nucleic Acids Res. 2016;44(D1): 279–85. 10.1093/nar/gkv1344 26673716 PMC4702930

[44] Finn RD, Clements J, Eddy SR. HMMER web server: interactive sequence similarity searching. Nucleic Acids Res. 2011;39(Web Server issue): W29–W37. 10.1093/nar/gkr367 21593126 PMC3125773

[45] Ashburner M, Ball CA, Blake JA, Botstein D, Butler H, Cherry JM, et al Gene Ontology: tool for the unification of biology. Nat Genet. 2000;25: 25 10.1038/75556 10802651 PMC3037419

[46] Grabherr MG, Haas BJ, Yassour M, Levin JZ, Thompson DA, Amit I, et al Full-length transcriptome assembly from RNA-Seq data without a reference genome. Nat Biotechnol. 2011;29: 644 10.1038/nbt.1883 21572440 PMC3571712

[47] Emms DM, Kelly S. OrthoFinder: solving fundamental biases in whole genome comparisons dramatically improves orthogroup inference accuracy. Genome Biol. 2015;16(1): 157 10.1186/s13059-015-0721-2 26243257 PMC4531804

[48] Maere S, Heymans K, Kuiper M. BiNGO: a Cytoscape plugin to assess overrepresentation of Gene Ontology categories in Biological Networks. Bioinformatics. 2005;21(16): 3448–9. 10.1093/bioinformatics/bti551 15972284

[49] Shannon P, Markiel A, Ozier O, Baliga NS, Wang JT, Ramage D, et al Cytoscape: A software environment for integrated models of biomolecular interaction networks. Genome Res. 2003;13(11): 2498–504. 10.1101/gr.1239303 14597658 PMC403769

[50] Smit A, Hubley R, Green P. RepeatMasker Open 4.0. 2013–2015 Available from: http://www.repeatmasker.org.

[51] Bao W, Kojima KK, Kohany O. Repbase Update, a database of repetitive elements in eukaryotic genomes. Mobile DNA. 2015;6(1): 11 10.1186/s13100-015-0041-9 26045719 PMC4455052

[52] Sekino M, Yamashita H. Mitochondrial DNA barcoding for Okinawan oysters: a cryptic population of the Portuguese oyster *Crassostrea angulata* in Japanese waters. Fisheries Sci. 2012;79(1): 61–76. 10.1007/s12562-012-0577-2

[53] Ghiselli F, Iannello M, Puccio G, Chang PL, Plazzi F, Nuzhdin SV, et al Comparative transcriptomics in two bivalve species offers different perspectives on the evolution of sex-biased genes. Genome Biol Evol. 2018;10(6): 1389–402. 10.1093/gbe/evy082 29897459 PMC6007409

[54] Lynch M, Conery JS. The origins of genome complexity. Science. 2003;302(5649): 1401 10.1126/science.1089370 14631042

[55] Hinegardner R. Cellular DNA content of the Mollusca. Comparative Biochemistry and Physiology Part A: Physiology. 1974;47(2): 447–60. 10.1016/0300-9629(74)90008-5 4156206

[56] Arkhipova IR. Neutral theory, transposable elements, and eukaryotic genome evolution. Mol Biol Evol. 2018;35(6): 1332–7. 10.1093/molbev/msy083 29688526 PMC6455905

[57] Sotero-Caio CG, Platt RN, Suh A, Ray DA. Evolution and diversity of transposable elements in vertebrate genomes. Genome Biol Evol. 2017;9(1): 161–77. 10.1093/gbe/evw264 28158585 PMC5381603

[58] Evgen’ev MB, Zelentsova H, Shostak N, Kozitsina M, Barskyi V, Lankenau D-H, et al *Penelope*, a new family of transposable elements and its possible role in hybrid dysgenesis in *Drosophila virilis*. Proc Natl Acad Sci USA. 1997;94(1): 196 8990185 10.1073/pnas.94.1.196 PMC19282

[59] Murgarella M, Puiu D, Novoa B, Figueras A, Posada D, Canchaya C. A first insight into the genome of the filter-feeder mussel *Mytilus galloprovincialis*. PLoS ONE. 2016;11(3): e0151561 10.1371/journal.pone.0151561 26977809 PMC4792442

[60] Vera M, Bello X, Álvarez-Dios J-A, Pardo BG, Sánchez L, Carlsson J, et al Screening of repetitive motifs inside the genome of the flat oyster (*Ostrea edulis*): Transposable elements and short tandem repeats. Mar Genomics. 2015;24: 335–41. 10.1016/j.margen.2015.08.006 26341181

