# Supplementary figures and images for: Comparative De Novo transcriptome analysis of the Australian black-lip and Sydney rock oysters reveals expansion of repetitive elements in Saccostrea genomes
Source: PLoS One. 2018 Oct 25;13(10):e0206417. doi: 10.1371/journal.pone.0206417 (PMC6201952; doi:10.1371/journal.pone.0206417)

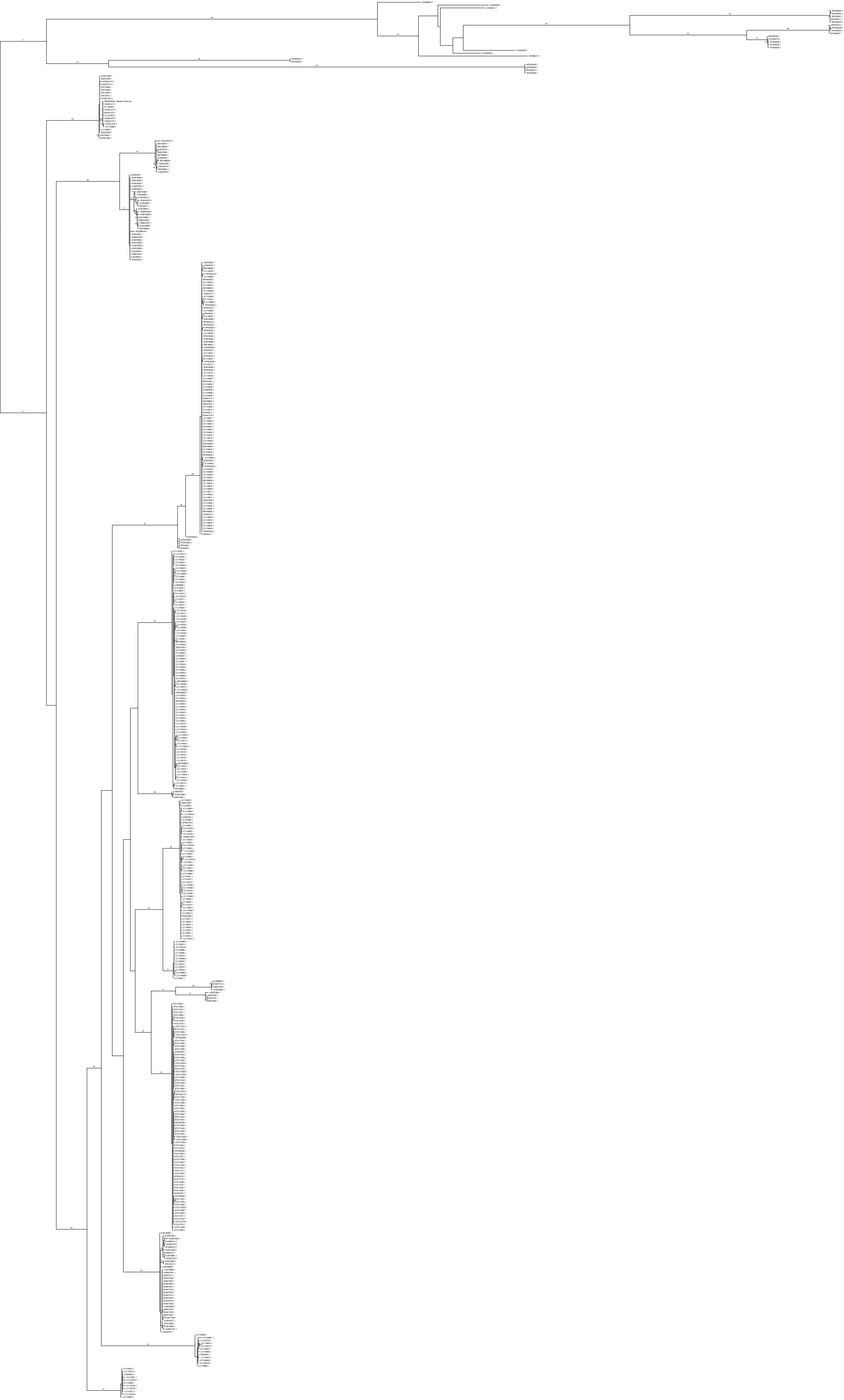

Supplement: S1 Fig — (PDF) [file pone.0206417.s002.pdf]
